# Promoting the wellbeing of the whole person: a within-subjects mixed-methods study exploring the effects of the flourishing intervention among individuals with depressive symptoms

**DOI:** 10.3389/fpsyt.2025.1532843

**Published:** 2025-06-19

**Authors:** Camilla Casaletti Braghetta, Juliane Piasseschi de Bernardin Gonçalves, Willyane de Andrade Alvarenga, Richard G. Cowden, Clarice Gorenstein, Giancarlo Lucchetti, Homero Vallada

**Affiliations:** ^1^ Department & Institute of Psychiatry, Faculdade de Medicina da Universidade de Sao Paulo, Sao Paulo, Brazil; ^2^ Centro Universitário Santo Agostinho, Teresina, Brazil; ^3^ Human Flourishing Program, Institute for Quantitative Social Science, Harvard University, Cambridge, MA, United States; ^4^ Department of Epidemiology, Harvard T.H. Chan School of Public Health, Boston, MA, United States; ^5^ Instituto e Departamento de Psiquiatria Laboratório de Investigação Médica-23 (LIM-23), Faculdade de Medicina da Universidade de São Paulo, São Paulo, Brazil; ^6^ Departamento de Farmacologia, Instituto de Ciências Biomédicas da Universidade de São Paulo, Sao Paulo, Brazil; ^7^ Department of Medicine, School of Medicine, Federal University of Juiz de Fora, Juiz de Fora, Brazil

**Keywords:** flourishing, wellbeing, depressive symptoms, life satisfaction, quality of life, spirituality

## Abstract

**Objective:**

This study examines the effect of the Flourishing Intervention on depressive symptoms and wellbeing among individuals with moderate to moderately severe depressive symptoms. The study also seeks to understand participants’ experiences, acceptability, and satisfaction with the intervention.

**Methods:**

A quasi-experimental pre-post mixed-methods design was used, incorporating an embedded approach in which descriptive qualitative data complemented quantitative data. The Flourishing Intervention consisted of a 12-week online group-based program, with each session lasting approximately 90 minutes. The study included 98 participants (18–59 years) with moderate to moderately severe depressive symptoms on the Patient Health Questionnaire-9 (PHQ-9), who had completed elementary school or better, resided in São Paulo, and had internet access. All participants were assessed immediately before and after the intervention using the PHQ-9, the Beck Depression Inventory-II (BDI-II), and a range of secondary outcome measures.

**Results:**

Evidence supported improvements in depressive symptoms postintervention (d=-1.14 for PHQ-9 and d=-1.24 for BDI-II). Positive postintervention changes were also observed for anxiety symptoms, personal flourishing, spirituality, quality of life, religious/spiritual coping, social support, happiness, gratitude, forgiveness, and life satisfaction.

**Conclusion:**

The Flourishing Intervention has the potential to be an effective approach for adults with depressive symptoms. It provides support for the idea that a multidimensional intervention focused on promoting whole-person functioning can alleviate these symptoms. Lessons learned from this study can be used to guide evaluation strategies for a controlled trial, which is an important next step in research on this intervention.

**Clinical trial registration:**

https://ensaiosclinicos.gov.br/rg/RBR-776skr9, identifier RBR-776skr9.

## 1 Introduction

The World Health Organization estimates that more than 30 million people are affected by depression, which has been identified as a leading global cause of disability (1). Data from the Global Burden of Disease in Brazil indicate that depressive disorders account for a substantial burden of disability in the country, with significant impacts on quality of life, functional status, and personal relationships (2).

Although there are several treatment options for depressive symptoms, including both pharmacological (e.g. antidepressants, mood stabilizers, atypical antipsychotics) and non-pharmacological approaches (e.g. psychotherapy, such as cognitive-behavioral therapy and interpersonal therapy), many people only experience partial improvements and/or short-term gains from such treatments (3). Many of these treatments come with high costs and need to be provided by qualified professionals, which has prompted a shift toward strengthening local capacity for mental health interventions and prioritizing cost-effective treatment options (4). High-cost treatments are critical in developing contexts, such as Brazil, where many socio-structural challenges (e.g. widespread poverty, high unemployment, unequal access to healthcare) can make it difficult for people to access treatment. Although Brazil has a public healthcare system, many people face significant barriers to accessing adequate mental health treatment (5).

Most mental health treatments take a symptomatic approach rooted in traditional psychology, focusing on reducing depressive symptoms rather than providing supportive tools and resources for maintaining improvements and preventing future depressive episodes (3). Positive psychology arose as the antithesis to this symptom-focused approach (6). It is oriented toward the positive aspects of human experience and applies a strengths-based lens, emphasizing “what is right” with a person (7).

Evidence concerning the effectiveness of positive psychology interventions (PPIs) seems promising. Previous meta-analyses (8, 9) have provided evidence suggesting that some interventions may be just as effective at reducing depressive symptoms as other mainstream treatment approaches, such as cognitive-behavioral therapy, physical activity, relaxation, or meditation training. However, most PPIs apply a componential approach by targeting isolated specific thoughts, emotions, or behaviors, rather than a holistic approach that considers the person as a whole (10). As one example, Chaves et al. (11) applied a multicomponent strategy for women with major depression by focusing on happiness, gratitude, positive emotions, acceptance, and optimism. They found that the intervention group improved depressive symptoms with an effect size of d=-0.54.

Delivering online interventions offers several advantages for individuals experiencing depressive symptoms (12). These include greater accessibility, flexibility, and overcoming logistical barriers such as scheduling conflicts and geographic limitations (13). Moreover, web-based formats can enhance privacy and reduce stigma, which may encourage participant engagement (13). Recent studies have shown that online interventions can be effective in promoting psychological wellbeing, particularly in populations with symptoms of depression, anxiety, or burnout, by allowing individuals to access support from any location and at a pace that is convenient for them (12).

In recent years, an expanded vision for human wellbeing has emerged with flourishing as a central aim. The concept of flourishing might be referred to as “the relative attainment of a state in which all aspects of a person’s life are good, including the contexts in which that person lives” (14, p. 38). While this expansive notion of human wellbeing has begun to change how scholars study wellbeing empirically (15), its impact on shaping approaches to promoting wellbeing has been more limited.

The main purpose of the present study is to examine the effect of an intervention to promote flourishing aspects (i.e. the Flourishing Intervention) on individuals with moderate to moderately severe depressive symptoms. As secondary outcomes, this study also seeks to explore the effect of the intervention on various secondary outcomes (e.g., quality of life, personal flourishing, religiosity/spirituality), as well as understanding the experiences, acceptability, and satisfaction of participants with the intervention. The intervention is expected to produce improvements in the primary and secondary outcomes.

## 2 Materials and methods

### 2.1 Study design and period

A quasi-experimental pre-post mixed-methods study was conducted from July 2022 to May 2024. The assessment of post-intervention outcomes followed an embedded design, integrating quantitative measures as the central evaluative tool with descriptive qualitative data exploring participants’ experiences to enrich understanding and identify potential explanations for quantitative results.

The research was approved by the Research Ethics Committee of the School of Medicine of the University of São Paulo, Brazil, under approval number CAAE: 52554221.4.0000.0068. It was also pre-registered on the Brazilian Clinical Trials Registration Platform under protocol number RBR-776skr9. All participants provided written informed consent.

### 2.2 Participants and eligibility criteria

To be eligible for participation, individuals needed to be between 18 to 59 years old, have scored moderate to moderately severe depressive symptoms (a score of 10 to 19 on the PHQ-9 scale) (16) at screening, have completed at least elementary school, be living in Sao Paulo, and have access to the internet. At baseline, those who had severe symptoms (scored above 19 on the PHQ-9) or scored positive on the suicidal ideation item on the PHQ-9 at baseline were excluded and referred to qualified professionals for care.

Adults aged 18 to 59 years were targeted, as they fall within the working age population, where depressive symptoms can result in notable functional impairment (17).

### 2.3 Setting and recruitment

The study was conducted entirely online. Recruitment was carried out through advertisements on the social media platforms of the São Paulo Clinics Hospital and via the professional networks of the team members. The notification included the study objectives, format, and duration of participation. Those interested were directed to a link to complete the recruitment and screening form containing sociodemographic questions, the PHQ-9, and a measure of gratitude (see Instruments section).

### 2.4 Intervention

The Flourishing Intervention used in this study takes a multidimensional approach to human wellbeing (18–20). It was designed through an evidence based development process (21), guided by a proposed conceptual framework for flourishing (18) and refined using expert consensus. The protocol was built in four phases: i) a literature review, ii) design of 12 structured group sessions, iii) expert evaluation using semi-structured questions, and iv) an e-Delphi technique involving an expert panel composed of PhD-level specialists in positive psychology, psychiatry, and spirituality (21). After a three-round process, a consensus was reached for the intervention protocol items. This process ensured the intervention’s conceptual coherence and practical feasibility.

The final version of the Flourishing Intervention is a 12-week online group based intervention, with each session lasting approximately 90 minutes. Session goals are to promote individual reflection using various strategies, such as group discussions, writing exercises, guided meditation exercises, sharing of videos and songs about the topic of the sessions, and reflective moments.

The following topics were addressed: (i) completion of the questionnaires and presentation of the program; (ii) mental and physical health, virtues and character strengths; (iii) love and gratitude; (iv) acts of kindness and volunteering; (v) happiness; (vi) family, friends, and community; (vii) forgiveness and compassion; (viii) resilience; (ix) spirituality and inner connection; (x) purpose and meaning of life; (xi) imagining the “best possible future” and flourishing; (xii) and program completion (see **Supplementary Material 1**).

### 2.5 Intervention providers

Two healthcare professionals conducted each session to provide content, delivery, and manage audiovisual resources. The provider’s team included professionals with clinical experience from different healthcare fields: nine psychologists, three social workers, one physiotherapist, and one occupational therapist. All providers underwent standardized training prior to implementation. The training was organized by the research team and consisted of 10 hours divided into two components: a theoretical module and a simulated role-play module. In the theoretical module, video lectures were provided by experts in relevant areas of the intervention. The simulation module included supervised role-play sessions for each provider. A continuous communication channel was established throughout the study, enabling providers to receive ongoing support and guidance from the training team.

In addition to the intervention team, two researchers (a nurse and an occupational therapist) conducted the post-intervention online focus groups. These researchers received separate training focused on qualitative methodology, including procedures for participant engagement, transcription quality, codebook development, and the ethical management of emotional risks. Clear protocols, including referral pathways, were established for identifying and addressing participant distress.

### 2.6 Procedure

The research team contacted eligible individuals who completed a recruitment form to participate in the study. Each participant was contacted via phone, email, or WhatsApp^®^ messages. Members of the research team provided details about the research and assessed participants’ interest and availability to proceed according to the scheduled days and times for the groups. Up to three contact attempts were made with each potential participant. Those who declined or were unavailable were excluded from the study. Enrolled participants were then assigned to the intervention.

Participants who were enrolled in the intervention completed the baseline assessment that included all measures presented in the Instruments section (with a duration of approximately 40–60 minutes). Since several groups were planned to run simultaneously on different days of the week, participants were randomly allocated to one of these groups. To obtain a balanced proportion of males and females and to avoid groups with only females, stratified randomization was performed using the estimated prevalence of depression in the Brazilian population, resulting in a ratio of 2 females to 1 male (2).

The intervention was delivered via the Zoom Meetings^®^ platform, with group sizes averaging 10 participants. Participants included in the study were invited to attend an initial session where the providers explained the group’s operational rules. At the end of this session, participants were invited to complete the baseline assessment on RedCap^®^. The informed consent form was presented on the first page of the survey.

The intervention was conducted weekly for over 12 sessions. It was determined that participants should complete at least 60% of the sessions to benefit from the intervention and to complete the study. Participating in at least 50-60% of the sessions has been considered necessary to obtain significant clinical benefits in group therapies, such as cognitive-behavioral therapy (3). Participants were reassessed immediately after the end of the intervention (approximately 40–60 minutes). Individuals who missed more than 60% of the sessions withdrew from the intervention or could not be re-contacted for follow-up were excluded from the analyses.

All participants were invited to participate in the qualitative study immediately upon completion of the intervention. Focus group interviews were conducted remotely via the Zoom Meetings^®^ platform with groups of no more than 10 people (lasting 60–90 minutes). Two trained members of the research team, who had no access to intervention during the study, conducted the interviews (see **Supplementary Material 2**). Focus groups were audio-recorded and transcribed verbatim.

Participants who dropped out of the study were also invited to an interview to understand their initial expectations of participating in the study, reasons for leaving, and feedback on the intervention to inform session improvements. Those who agreed had their interviews recorded for later transcription and analysis.

### 2.7 Instruments

The assessment included the following measures that required about 40 to 60 minutes to complete.

Primary outcomes:

- Depressive symptoms were assessed using the PHQ-9 (22), a 9-item self report screening tool (with scores ranging from 0 to 27) for identifying adults in the general population who are at higher risk of experiencing major depressive episodes. Items are rated using a four-point Likert scale from 0 (not at all) to 3 (nearly every day), based on symptom frequency over the past two weeks. PHQ-9 scores below 5 suggest the absence of a depressive disorder; scores from 5–9 predominantly represent patients with subthreshold depression. Scores from 10–14 represent a spectrum of individuals with mild to moderate depressive symptoms; scores from 15-19 suggest moderate to severe symptoms; and scores of 20 or higher usually indicate major depression. The original PHQ-9 Portuguese validation study demonstrated good internal consistency (α=0.89) (22).
- The Beck Depression Inventory-II (BDI-II) was also used to assess depressive symptoms (23). The BDI-II is a well-validated self-report measure of depressive symptoms, and it has been used extensively in both clinical and nonclinical populations to estimate the prevalence of cases of depression in various populations. This scale has 21 items. Each item consists of four statements rated from 0 to 3 varying by item (e.g., from 0 *I do not feel sad* to 3 *I am so sad or unhappy that I can’t stand it*), reflecting increasing symptom severity, with participants selecting the statement that best describes how they have been feeling over the past two weeks. Scores range from 0-63, where 0–9 indicates that the individual is not depressed, a score of 10–18 suggests mild to moderate depression, a score of 19–29 indicates moderate to severe depression, and a score of 30–63 suggests severe depression. BDI-II was validated in Portuguese with an excellent internal consistency (α=0.88) (23).

Secondary outcomes:

- Anxiety was measured using the Generalized Anxiety Disorder 7 (GAD-7) (24), which is a 7-item self-report questionnaire with a score range of 0–21. Items are rated using a four-point Likert scale from 0 (not at all) to 3 (nearly every day), reflecting symptom frequency over the past two weeks. Higher scores reflect greater severity of anxiety. Scores of 0–4 suggest minimal anxiety, 5–9 indicate mild anxiety, 10–14 moderate anxiety, and 15–21 indicate severe anxiety. Prior research on the Portuguese version of the GAD-7 suggests the measure has excellent internal consistency (α=0.91) (24).
- Common mental health symptoms were assessed using the Self-Reporting Questionnaire 20 (SRQ-20) (25), which is a screening tool with a score range of 0–20 that includes depressive symptoms, anxiety, and psychosomatic complaints. Items are answered using a dichotomous response format (yes/no). Scores of ≥7 on the SRQ-20 suggest the presence of common mental disorders in community samples. Higher scores reflect greater emotional distress, including symptoms of depression, anxiety, and somatization. In a previous study, this Portuguese validated instrument has shown high internal consistency (α=0.80) (25).
- Quality of life was measured by the World Health Organization Quality of Life Assessment (WHOQOL-BREF) (26), which contains 26 items. Of those, 24 items comprise the four domains of physical health, psychological health, social relationships, and environment. The other two items measure overall quality of life and general health. Items are rated using a five-point Likert scale, varying anchors by item (e.g., from 1 *very dissatisfied* to 5 *very satisfied*). Domain scores are transformed to range from 0–100, with higher scores indicating better quality of life in each domain. The Portuguese version of the instrument has shown satisfactory internal consistency in its validation study (α=0.69-0.91 across domains) (26).
- Personal flourishing was assessed using the Secure Flourish Index (27). This instrument assesses six domains of human functioning: happiness and life satisfaction, mental and physical health, meaning and purpose, character and virtue, close social relationships, and financial and material stability. A total score is obtained by summing the scores from the twelve questions in all six domains, resulting in a score from 0-120. Items are rated using an 11-point response scale from 0 (not at all) to 10 (completely). Higher scores imply higher levels of personal flourishing. The Portuguese validation study has shown good internal consistency (α=0.86) (28).
- Religiosity was assessed with the DUREL (29), which is a 5-item measure assessing three dimensions of religiosity: organizational religious activity, non-organizational religious activity, and intrinsic religiosity. Higher scores indicate higher levels of organizational, non-organizational, or intrinsic religiosity. The three domains are analyzed separately. Items are rated using a 5-point or 6-point response scale varying by item (e.g., from 0 *definitely not true* to 5 *definitely true*). Appropriate internal consistency was observed for the intrinsic religiosity dimension of this scale in its Portuguese language validation study (α=0.758) (30).
- Religious coping was measured using the Brief RCOPE (31), a 14-item measure divided into two subscales corresponding to positive and negative religious/spiritual coping. Items are rated using a four-point response scale from 1 (never) to 4 (very often). In the Portuguese validation study, the subscales demonstrated excellent internal consistency (α=0.98 for positive religious coping; α=0.86 for negative religious coping) (31).
- Spirituality was assessed with the Attitudes Related to Spirituality Scale (ARES) (32), an 11-item measure (score range of 11-55) that assesses aspects inherent to spirituality and was developed originally in the Portuguese language. Items are rated using a five-point response scale from 1 (strongly disagree) to 5 (strongly agree). Higher scores suggest higher levels of spirituality. The measure showed excellent internal consistency in the development study (α=0.98) (32).
- Gratitude was assessed using the Brazilian Gratitude Scale (B-GRAT- 20) (33), a 13-item measure of gratitude for life experiences. Items are rated using a five-point response scale from 1 (strongly disagree) to 5 (strongly agree). Scores range from 13 to 65, with higher scores indicating greater gratitude. Internal consistency was high in the Portuguese validation study (α=0.95) (33).
- Compassion was measured using the Santa Clara Compassion Brief Scale (34), a 5-item scale in which items are rated from 1 (not at all true of me) to 7 (very true of me). Scores range from 5 to 35, with higher scores indicating greater compassion. In prior research, internal consistency for the measure has been good (α=0.84) (34).
- Altruism was assessed with the Altruism Scale (35), a 20-item measure in which participants indicate how often they have engaged in altruistic behaviors. Items are rated using a five-point response scale from 1 (never) to 5 (very often). Higher total scores indicate more frequent engagement in altruistic behaviors. The measure exhibited good internal consistency in the Portuguese validation study (α=0.83) (35).
- Volunteering was assessed by asking participants whether they had done any volunteering in the last month and how many hours they had volunteered.
- Happiness was measured using the Subjective Happiness Scale (SHS) (36), a four-item instrument assessing global subjective happiness through statements where participants either evaluate themselves or make comparisons. Items are rated using a seven-point response scale, with anchor points tailored to each item (e.g., from 1 less happy to 7 more happy). Scores range from 4 to 28, with higher scores reflecting greater subjective happiness. In a prior Portuguese validation study, the SHS showed acceptable internal consistency (α=0.77) (36).
- Social support was assessed through the Multidimensional Scale of Perceived Social Support (37), an instrument developed to assess perceived social support. Items are rated using a seven-point response scale from 1 (very strongly disagree) to 7 (very strongly agree). Scores range from 12 to 84, with higher scores indicating greater perceived social support from family, friends, and significant others. The Portuguese version of the measure demonstrated excellent internal consistency in a prior validation study (α=0.93 for friends, α=0.91 for family, and α=0.90 for significant others) (37).
- Forgiveness was measured using the Heartland Forgiveness Scale (HFS) (38), an 18-item measure with items rated using a 7-point scale with a seven-point response scale from 1 (almost always false of me) to 7 (almost always true of me). Total scores range from 18 to 126, with higher scores indicating a greater tendency to forgive. In prior validation research examining the HFS in Portuguese, acceptable internal consistency has been reported (α=0.88) (38).
- Resilience was assessed using the Resilience Scale (RS) (39), which consists of 25 items rated on a 7-point Likert scale rated on a seven-point Likert scale from 1 (strongly disagree) to 7 (strongly agree), with total scores ranging from 25 to 175. Higher scores indicate greater levels of resilience. The RS has been shown to have good internal consistency in prior research examining its validity in Portuguese (α=0.83) (39).
- Purpose in Life was measured using the Purpose in Life Test (PLT) (40), a scale comprising 22 items with anchors tailored to each item (e.g., from 1 no sense of purpose to 7 clear sense of purpose). Scores range from 22 to 154, with higher scores indicating a greater sense of purpose and meaning in life. The Portuguese version of the PLT has demonstrated acceptable internal consistency in previous research (α=0.76) (40).
- Satisfaction with Life was assessed using the Satisfaction with Life Scale (SLS) (41), a five-item measure in which items are rated using a seven point Likert scale from 1 (strongly disagree) to 7 (strongly agree). Total scores range from 5 to 35, with higher scores reflecting greater life satisfaction. Prior research with the Portuguese version of the SLS has reported high internal consistency for the measure (α=0.88) (41).
- Sociodemographic and general health information, including age, sex, education, religion, race, family income, current medical treatment and medications, and ongoing psychotherapeutic and complementary treatments, was also collected from participants.

All instruments used in this study were previously validated or culturally adapted for the Brazilian population. The corresponding references cited above provide detailed psychometric properties for each measure.

### 2.8 Sample calculation

Sample size estimation was performed using G*Power 3.1. The Minimally Relevant Clinical Difference (MCID) of the PHQ-9 score for depressive symptoms was set to a 5-point change after the intervention by a previous study (42). Based on this 5-point MCID and adopting a standard deviation of 8.00, the calculated effect size was 0.62. Using the sample size calculation for a difference between two dependent means and specifying a two-tailed test, alpha of 0.05, and power (1-Beta) of 0.95, the minimum sample size estimated was 36 participants. Given the 9-month follow-up period for this study, we anticipated a dropout rate of approximately 30%-40%, consistent with attrition rates commonly observed in longitudinal interventions involving individuals with depressive symptoms. For instance, studies have reported attrition rates up to 36% in clinical trials and up to 65% in naturalistic settings during the first 12 weeks of treatment for depression (43).

### 2.9 Statistical analyses

All quantitative data were collected using RedCap^®^ and exported to STATA 13. First, a descriptive analysis was used to show absolute and relative frequencies, means, and standard deviations. Then, inferential analysis was conducted to compare baseline to post-intervention changes in the primary and secondary outcomes. Paired samples *t*-tests were used for continuous variables, and McNemar tests were used for categorical variables. An independent samples *t*-test was also used to compare those who completed the intervention with those who dropped out. Effect sizes were also generated using Cohen’s *d* coefficient.

A *p*<0.05 was adopted as significant, and a 95% confidence interval was set.

#### 2.9.1 Qualitative analyses

Inductive qualitative content analysis was used to analyze qualitative data obtained from the focus groups (44). Two researchers, an occupational therapist and a nurse, who conducted the focus groups, checked data integrity after transcribing the interviews. They developed a codebook with an initial coding scheme concerning: (1) the perceived impact of the intervention (symptoms of depression, changes in behavior, lifestyle, relationships, emotions and thoughts of the participants); (2) the facilitators and barriers to engagement in the sessions; and (3) suggestions for improving the intervention. NVivo^®^ software was used to organize qualitative data for coding and creating key categories. The researchers continually discussed divergent opinions about categorization of responses and the adequacy of the analysis.

A reflexivity process was used to examine how the researchers’ professional backgrounds, the timing of data collection immediately following the intervention and the power dynamics between participants and researchers may have influenced the context in which data were collected. Trustworthiness and credibility were ensured through independent and team analysis by researchers without access to the quantitative results. Participant quotes were used to illustrate the findings (excerpts were anonymized). The Standards for Reporting Qualitative Research (SRQR) were used as a guide to report the qualitative component of the research (45).

## 3 Results

### 3.1 Quantitative results

**Figure 1:**
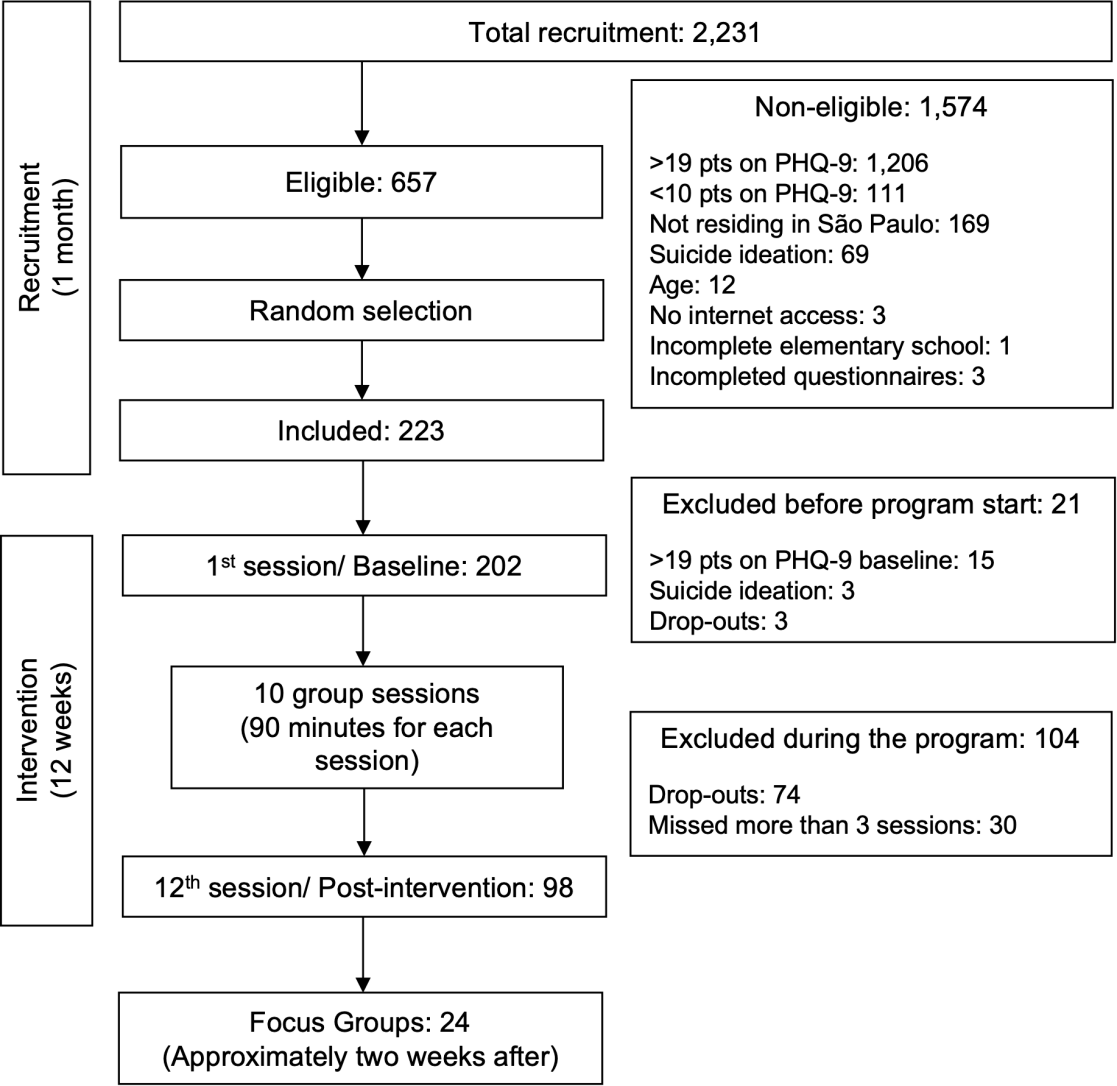
Flow diagram of participant eligibility and inclusion in the study.

**Figure 1** shows the flowchart of the recruitment and participant selection process. Of the 2,231 individuals screened, 1,574 were excluded for not meeting the inclusion criteria. The main reasons for exclusion in this stage were scoring above 19 on the PHQ-9 (n=1,206), scoring below 10 on the PHQ-9 (n=111), and scoring positive on the suicidal ideation item (n=69). Of the 657 eligible individuals, a sample of 223 individuals was randomly assigned to the concurrent groups receiving the intervention, as described in the previous section. Among those included, 18 individuals scored above 19 on the PHQ-9 or presented suicidal ideation at baseline and were excluded (individuals with severe symptoms were contacted and referred for evaluation and treatment with a psychiatrist in primary healthcare), and three dropped out of the study before the intervention started due to personal reasons. Thus, a total of 202 participants started the intervention. During the intervention, 74 individuals dropped out at different stages, and 30 participants missed more than four sessions. They were removed from the analyses, resulting in a sample of 98 participants (48.5% retention).

**Table 1** presents the baseline characteristics of the participants who completed the study compared to those who did not. The sample of participants was predominantly female (69.8%), with a mean age of 41.45 years (SD=9.21), reported their marital status as single or separated (54.5%), had completed a university degree or higher (79.7%), and identified ethnically as white (73.8%). Most participants were not engaged in ongoing psychiatric care (70.3%) but were receiving psychiatric medication (61.4%). The mean overall score on the PHQ-9 was 15.15 (SD=2.55) at screening and 13.52 (SD=3.77) at baseline. There was a significant difference only for age between the dropout group and those who completed the intervention (completers: M=43.10, SD=0.90 vs. dropouts: M=39.89, SD=0.90, p=0.012). We did not find evidence of baseline differences between the two groups in any other measure.

**Table 1:** Sociodemographic characteristics and baseline outcomes among individuals who completed the Flourishing Intervention and those who dropped out.

Variables	Total (n=202)		Completed (n=98)		Dropouts (n=104)		
*Sociodemographic*							
*Sex †*	n	%	n	%	n	%	p-value
Male	61	30.2	27	27.5	34	32.7	0.426
Female	141	69.8	71	72.4	70	67.3	
*Marital status †*	n	%	n	%	n	%	p-value
Married/living together	92	45.5	49	50	61	41.3	0.217
Single/separated/widow	110	54.5	49	50	43	58.6	
*Education †*	n	%	n	%	n	%	p-value
Below university degree	41	20.3	16	16.3	25	24	0.173
Completed university degree or higher	161	79.7	82	83.7	79	76	
*Race †*	n	%	n	%	n	%	p-value
White	149	73.8	73	75.5	76	73.1	0.820
Black/Brown/Others	53	26.2	25	25.5	28	29.9	
	Mean	SD	Mean	SD	Mean	SD	p-value
Age *	41.45	9.21	**43.10**	**0.90**	**39.89**	**0.90**	**0.012**
*Wellbeing*							
	Mean	SD	Mean	SD	Mean	SD	p-value
Personal Flourishing*	62.28	16.71	62.81	17.52	61.78	15.98	0.663
Satisfaction with life *	18.76	6.47	19.27	6.25	18.28	6.66	0.260
*Physical Health*							
*Other medical treatment †*	n	%	n	%	n	%	p-value
Yes	67	33.2	32	32.6	35	33.6	0.880
No	135	66.8	66	67.3	69	66.3	
	Mean	SD	Mean	SD	Mean	SD	p-value
Quality of life physical *	53.06	13.52	52.29	13.34	53.77	13.72	0.438
*Mental Health*							
*Psychiatric treatment †*	n	%	n	%	n	%	p-value
Yes	60	29.7	31	31.6	29	27.9	0.560
No	142	70.3	67	68.4	75	72.1	
*Psychiatric medication †*	n	%	n	%	n	%	p-value
Yes	124	61.4	62	63.3	62	59.6	0.594
No	78	38.6	36	36.7	42	40.4	
*Weekly psychotherapy †*	n	%	n	%	n	%	p-value
Yes	68	33.7	32	32.6	36	34.6	0.768
No	134	66.3	66	67.3	68	65.4	
	Mean	SD	Mean	SD	Mean	SD	p-value
Depression PHQ-9 baseline *	13.52	3.77	13.46	4.00	13.58	3.56	0.826
Depression BDI *	25.29	8.49	25.07	8.59	25.50	8.44	0.715
Common Mental Health symptoms*	11.24	3.30	11.14	3.40	11.33	3.22	0.679
Anxiety *	10.08	4.67	9.87	4.23	10.27	5.07	0.543
Quality of life psychological *	45.50	13.64	46.34	13.96	44.71	13.36	0.397
*Social and Environment*							
	Mean	SD	Mean	SD	Mean	SD	p-value
Quality of life environment *	43.27	19.15	44.39	18.14	42.23	20.09	0.424
Quality of life social relationships *	56.59	14.98	56.47	14.21	56.71	15.74	0.914
Social support total *	55.00	15.84	56.71	15.42	53.38	16.13	0.170
Social support family *	17.12	6.44	17.71	6.05	16.57	6.78	0.231
Social support friends *	17.82	6.67	18.59	5.99	17.10	7.20	0.146
Social support others *	20.04	6.26	20.40	6.10	19.70	6.42	0.495
*Religiosity and Spirituality*							
	Mean	SD	Mean	SD	Mean	SD	p-value
Spirituality *	43.22	12.26	43.52	12.14	42.94	12.43	0.739
Organizational religiosity *	2.94	1.50	3.04	1.53	2.85	1.46	0.381
Non-organizational religiosity *	4.15	1.96	4.41	1.89	3.90	2.01	0.059
Intrinsic religiosity *	9.74	3.77	9.81	3.64	9.68	3.90	0.770
Positive religious coping *	21.54	8.89	21.08	8.73	21.99	9.06	0.500
Negative religious coping *	30.92	5.24	30.96	5.44	30.87	5.07	0.939
*Virtues and Values*							
	Mean	SD	Mean	SD	Mean	SD	p-value
Volunteering *	0.25	0.43	0.28	0.45	0.23	0.42	0.312
Meaning of life *	89.76	15.30	89.39	14.64	90.11	15.96	0.764
Resilience *	112.50	19.50	112.55	18.07	112.47	20.84	0.922
Altruism *	51.26	13.07	51.50	12.49	51.54	13.66	0.755
Happiness *	16.26	3.83	16.27	4.13	16.25	3.56	0.952
Gratitude *	28.10	6.28	28.47	5.50	27.75	6.94	0.380
Forgiveness total *	77.31	15.15	77.24	13.43	77.38	16.67	0.997
Compassion *	28.21	5.76	28.74	5.01	27.71	6.38	0.175

N, sample; %=percentage; SD, standard deviation; †, McNemar test; *, unpaired *t*-test. Significant differences are highlighted in bold.

**Table 2** reports baseline and post-intervention outcome comparisons. There was a significant change in scores of PHQ-9 and BDI-II. On PHQ-9, the average score before the intervention was 13.47 (SD=4.00), while after the intervention, the average was 8.35 (SD=4.92), suggesting a large effect size (d=1.14). On the BDI-II, the average score before the intervention was 25.07 (SD=8.59), whereas after the intervention, the average was 13.49 (SD=10.01), also suggesting a large effect size (d=-1.24).

**Table 2:** Results for participants who completed the Flourishing Intervention.

Variables	Pre-intervention (n=98)		Post-intervention (n=98)			
*Wellbeing*						
	Mean	SD	Mean	SD	p-value	ES
Personal Flourishing *	**62.82**	**17.52**	**77.02**	**18.04**	**0.001**	**0.80**
Satisfaction with life *	**19.27**	**6.25**	**22.47**	**6.52**	**0.001**	**0.50**
*Physical Health*						
*Other medical treatment †*	n	%	n	%	p-value	
Yes	29	36.25	33	41.25	0.503	
No	51	63.75	47	58.75		
	Mean	SD	Mean	SD	p-value	ES
Quality of life physical *	**52.29**	**13.34**	**61.00**	**15.38**	**0.001**	**0.69**
*Mental Health*						
*Psychiatric treatment †*	n	%	n	%	p-value	
Yes	28	35	25	31.25	0.375	
No	52	65	55	68.75		
*Psychiatric medication †*	n	%	n	%	p-value	
Yes	53	66.25	55	68.75	0.727	
No	27	33.75	25	31.25		
*Other medical treatment †*	n	%	n	%	p-value	
Yes	29	36.25	33	41.25	0.503	
No	51	63.75	47	58.75		
*Weekly psychotherapy †*	n	%	n	%	p-value	
Yes	**24**	**30**	**39**	**48.75**	**0.001**	
No	**56**	**70**	**41**	**51.25**		
	Mean	SD	Mean	SD	p-value	ES
Depression PHQ-9 *	**13.47**	**4.00**	**8.35**	**4.92**	**0.001**	**-1.14**
Depression BDI *	**25.07**	**8.59**	**13.49**	**10.01**	**0.001**	**-1.24**
Common Mental Health symptoms *	**11.14**	**3.41**	**6.65**	**4.31**	**0.001**	**-1.15**
Anxiety *	**9.88**	**4.23**	**5.96**	**4.52**	**0.001**	**-0.60**
Quality of life psychological *	**46.34**	**13.96**	**57.06**	**14.12**	**0.001**	**0.80**
*Social and Environment*						
	Mean	SD	Mean	SD	p-value	ES
Quality of life environment *	**44.39**	**18.14**	**54.42**	**19.81**	**0.001**	**0.53**
Quality of life social relations *	**56.47**	**14.21**	**62.18**	**14.16**	**0.001**	**0.54**
Social support total *	**56.71**	**15.42**	**62.83**	**14.01**	**0.001**	**0.41**
Social support family *	**17.71**	**6.05**	**19.49**	**6.40**	**0.001**	**0.28**
Social support friends *	**18.59**	**5.99**	**20.93**	**5.27**	**0.001**	**0.41**
Social support others *	**20.41**	**6.10**	**22.41**	**5.06**	**0.001**	**0.36**
*Religiosity and Spirituality*						
	Mean	SD	Mean	SD	p-value	ES
Spirituality *	**43.52**	**12.14**	**44.78**	**11.64**	**0.021**	**0.11**
Organizational religiosity *	3.04	1.54	3.02	1.50	0.820	0.01
Non-organizational religiosity *	4.42	1.89	4.47	1.93	0.667	0.03
Intrinsic religiosity *	**9.82**	**3.65**	**10.47**	**3.79**	**0.002**	**0.17**
Positive religious coping *	**21.08**	**8.73**	**23.55**	**9.14**	**0.001**	**0.28**
Negative religious coping *	**30.97**	**5.45**	**31.99**	**4.43**	**0.018**	**0.20**
*Virtues and Values*						
	Mean	SD	Mean	SD	p-value	ES
Volunterring *	0.28	0.45	0.33	0.47	0.158	0.09
Meaning of life *	**89.40**	**14.65**	**99.84**	**16.01**	**0.001**	**0.68**
Resilience *	**112.55**	**18.08**	**122.11**	**19.86**	**0.001**	**0.50**
Altruism *	51.5	12.49	52.78	12.81	0.142	0.10
Happiness *	**16.27**	**4.13**	**17.66**	**3.73**	**0.001**	**0.35**
Gratitude *	**28.48**	**5.50**	**30.31**	**5.33**	**0.001**	**0.34**
Forgiveness total *	**77.24**	**13.44**	**85.68**	**15.34**	**0.001**	**0.58**
Compassion *	28.74	5.01	29.17	5.10	0.282	0.08

N, sample; %, percentage; SD, standard deviation; ES, effect size; †, McNemar test; *, paired *t*-test. Significant differences are highlighted in bold.

For the secondary outcomes, the largest effect sizes were for personal flourishing (d=0.80) and common mental health symptoms (d=-1.15). Changes were significant for other variables, such as anxiety, spirituality, quality of life, both positive and negative religious coping, social support, happiness, gratitude, forgiveness, and satisfaction with life, but not for volunteering, religiosity, altruism, and compassion. Psychiatric treatment, psychiatric medication, and medical treatment did not change after the intervention, but there was an increase in weekly psychotherapy (30% before and 48% after the intervention, p=0.001).

### 3.2 Qualitative results

A total of 10 individuals who dropped out of the intervention agreed to participate in the qualitative component of the study. Among those who dropped out after the first session, reasons included scheduling conflicts due to changes at work (n=2) and health treatment necessity (n=1). The seven individuals who dropped out after two sessions reported various reasons, including difficulties in adjusting to a group format and a preference for individual care (n=3), disappointment that the program did not meet their expectations or align with the sequence they anticipated (n=2), and discomfort with sharing personal issues with the group (n=2).

Seven virtual focus groups were held after the intervention with those who completed it, totaling 24 participants (15 women and nine men). Each focus group lasted an average of 80 minutes. Based on the data from the focus groups, three themes were developed: “positive changes after participating in the intervention,” “motivation to join the intervention,” and “the acceptability of the intervention format.”

#### 3.2.1 Positive changes after participating in the intervention

Participants reported a variety of positive changes across psychological, emotional, behavioral, and social domains. Psychologically, many experienced a reduction in depressive symptoms, such as improved mood, greater levels of energy, less emotional reactivity, and reduced self-judgment, alongside meaningful changes in personal attitudes and a renewed understanding of spirituality.

*“And I’ve noticed that since then I haven’t had any more anxiety attacks. I used to have them a lot, it was a tightness in my chest, it was horrible, but still, wow, it was like that, I managed with everything I heard and everything, we even had a few, two or three sessions where we had moments of meditation and it was so good, that was ours! It was liberating” (Participant 2)*

Emotionally, participants described feeling lighter, calmer, more patient, and resilient. Improvements in self-care included adopting healthier routines, such as engaging in physical exercise, continuing psychotherapy, joining support groups, and dedicating more time to reading. Several individuals also reported increased motivation to pursue personal goals and finish lingering incomplete projects.

*“I managed to finish a course that I had been putting off for a long time to take the exams, I finished it last week when you weren’t here with me, I did it, I completed this course. So, now you’re asking, I completed these three months with you, I managed to complete the course that was pending” (Participant 20)*

Socially, many individuals experienced the group as a source of connection and support, and described improvements in communication, empathy, and appreciation for their social networks.

*“And then, I also feel like going out and meeting people, like the girls said. And just like (cited other Participant name), I also received positive feedback, right?” (Participant 5)*

Participants described making lifestyle changes due to the intervention, such as starting or increasing physical exercise, feeling motivated to continue psychotherapy, joining other support groups, and reading more. These changes were perceived as important steps toward improving their emotional wellbeing and maintaining the benefits experienced during the intervention.

*“I had a huge difficulty reading and I started to challenge myself to read a little bit each day and I started to enjoy reading more” (Participant 13)*

Other participants reported feeling more motivated to engage in personal projects and pursue meaningful goals after the intervention. Some described setting more personal goals and experiencing renewed focus and direction daily. They also shared the satisfaction of achieving personal milestones, such as adopting a pet or completing long-term goals that had previously been postponed.These actions were often framed as signs of regained autonomy and hope for the future.

*“I managed to finish a course that I had been putting off for a long time to take the exams, I finished it last week when you weren’t here with me, I did it, I completed this course. So, now you’re asking, I completed these three months with you, I managed to complete the course that was pending.” (Participant 20)*

#### 3.2.2 Motivation to join the intervention

Many participants reported that they were motivated to join the Flourishing Intervention because it was an opportunity to take care of themselves and improve different aspects of their lives, such as symptoms of depression and anxiety, physical health, work-related issues, and interpersonal relationships.

*“When I started the intervention, I was going through a difficult time. I was unemployed, having financial problems, and then I saw the ad. I think it was on Instagram, and precisely, it was something that I needed at the time too. I had never done anything like this, like therapy, psychological treatment … And then I said, “Ah, I’m going to do a test to see if I like it” (Participant 6)*

Other common reasons for joining the intervention centered on personal change or growth.

*“When I started, I was going through a phase where my therapist and my friends said that I had a lot to change, that I had a lot to look at myself, that I had to change for myself. And when I started, flourishing was exactly what I wanted for myself, you know, to flourish like that, like change and grow, and my expectation was to be able to look at myself more and I think that was met” (Participant 3)*

Additional information about the qualitative analysis can be found in **Table 3**.

**Table 3:** Participant perspectives on the acceptability of the intervention format.

Acceptability of the intervention format	Sample quotes
**Group format** Considered the group a support network Wished the group had continued Felt comfortable within the group First experience participating in a group	*“I think we are creating a community of exchange and also of security here, we feel safe in being able to exchange and comment on things and so on. So, I think that sometimes means we think, wow, it could go on a little longer.” (Participant 32)* *“I had some prejudice against this kind of activity, and maybe the fact that it was a group setting made it more comfortable for me. People welcomed me warmly, and in the end, I’ll finish with the same words I started with: I’m grateful for everything that happened.” (Participant 29)* *”I still really miss it, because there are days when you’re not doing so well, and others when you are—and that weekly meeting made a difference. You had activities that connected to your daily life, to things you’d been through, and you’d end up remembering them in the moment, in a way that was practical during the week. And that helped a lot in day-to-day life.” (Participant 22)*
**Weekday convenience** Monday Tuesday Thursday Friday is the least convenient day Once a week is best	*“I thought the format was great. Ninety minutes is a very good amount of time. I think one hour isn’t quite enough—some people want to* sp*eak, others do too. But if it goes beyond ninety minutes, we’re already more tired, less willing. And once a week is great too, because I think … you need time to digest things, right? You receive the information, and then you process it during the week.” (Participant 1)* *“I think Friday would be harder to get people together, you know? Everyone wants to go out. I think Friday would be the worst day. I don’t think there would be any problem with the other days.” (Participant 3)*
**Preference for online format** No difficulty with commuting Difficulty commuting due to illness Disadvantage of not being able to meet in person	*“I thought it was excellent, because if I had to travel, I would miss more. It would be more difficult, due to work, traffic, rain, etc. So, for me, I thought this format was the best.” (Participant 13)* *“Well, for me too … I really like the in person format. I enjoy getting to know people face-to-face, I think there’s something special about that human warmth. But … I admit that maybe I wouldn’t have been able to participate if it hadn’t been online.” (Participant 17)*

## 4 Discussion

To our knowledge, this is one of the first studies to examine the effect of a multidimensional wellbeing intervention that aligns with a holistic view of human flourishing (20, 46). Our results supported significant improvements in depressive symptoms after participating in the Flourishing Intervention. Positive changes were also observed for several secondary outcomes, with effect sizes ranging from small (e.g., compassion d=0.08) to very large (e.g., common mental health symptoms d=-1.15). Results from the qualitative analysis of focus groups with participants who completed the Flourishing Intervention corroborated the quantitative findings, showing that participants changed their psychological functioning and lifestyle, experienced more positive emotions, were motivated to participate in the intervention, and found engaging in the online approach easy. By extending beyond the conventional treatment model that concentrates primarily on addressing symptoms and illness and encouraging a more comprehensive perspective that emphasizes values, virtues, strengths, and positive emotions, our findings suggest that the Flourishing Intervention has potential implications for supporting people with depressive symptoms.

For the primary outcomes, we observed a considerable reduction in depressive symptoms after the intervention (d=-1.14 for the PHQ-9 and -1.24 for the BDI). These findings align with previous studies investigating multicomponent PPIs (9, 47, 48). For example, in one meta-analysis with 347 studies involving over 72,000 participants, Carr et al. (9) found that PPIs were effective at reducing depressive symptoms (g=-0.39), anxiety symptoms (g=-0.62), and stress (g=0.58), as well as increasing quality of life (g=0.48), character strengths (g=0.46), and wellbeing (g=0.39), as compared to controls. Carr and colleagues also discussed that multicomponent protocols that were longer in duration and offered in person rather than self-help intervention seemed to show better outcomes (9). These characteristics are similar to those used in the Flourishing Intervention, since it addresses multiple virtues and values using an online group approach with a 12-week duration.

Although we observed large effect sizes for depressive symptoms, comparing our findings with pre-post-intervention effect sizes reported in prior research is important because our study did not have a control group. We found four noteworthy studies with similar methodological designs and therapeutic approaches.

Asgharipoor et al. (49) worked with a sample of adults who had major depressive disorder using a 12-week group session protocol on positive psychotherapy. The topics discussed included themes related to cultivating a pleasant, engaged, and meaningful life. Changes from pre- to post-intervention resulted in an effect size of d=-1.50 for the BDI-II. In another study, Furchtlehner et al. (50) carried out a 14-week group session intervention provided by psychotherapists. Their study included individuals with mild to moderate depressive symptoms and covered topics such as character strengths, forgiveness, hope, and optimism. The effect size for pre- to post-intervention changes in depressive symptoms for those who received the intervention was d=-1.47.

Another intervention protocol called “Say Yes to Life”, proposed by Carr et al. (51), was implemented among a sample of adults diagnosed with major depressive disorder. It consisted of 20 sessions, each lasting 2 hours, conducted by psychologists and addressing topics such as forgiveness, resilience, relationships, and gratitude. The pre-to-post intervention effect size for the BDI-II was d=-2.12. Finally, Chaves et al. (11) conducted a study with a sample of women experiencing major depression or dysthymia. The protocol consisted of 10 group sessions, each lasting 2 hours, addressing happiness, gratitude, positive emotions, acceptance, and optimism. The effect size for changes in depressive symptoms from pre- to post-intervention was d=-0.54.

The effect sizes of those multicomponent PPIs are very similar to our findings on the BDI-II outcome. However, a few differences should be highlighted. Each prior study was conducted in person and was provided by a licensed mental health professional (typically psychologists). In contrast, the Flourishing Intervention was delivered entirely online by a trained provider who was not required to be a licensed mental health professional.

Another difference is that the Flourishing Intervention is grounded in an expanded view of flourishing. Although many PPIs cover a few different topics, important aspects such as physical health and spiritual beliefs are not commonly addressed. Applying a holistic approach to wellbeing (46), our findings highlight the potential utility of multidimensional interventions that target different dimensions of wellbeing to support people with depressive symptoms. This broader view of wellbeing has been supported by post-intervention improvements observed for the physical quality of life and some religious/spiritual outcomes, as well as the qualitative data with those who completed the Flourishing Intervention. After the intervention, many participants reported paying more attention to their wellbeing. Some reported changing their lifestyle, such as increasing their physical activity or social participation.

Some participants also reported changes in their perspective on spirituality, understanding it not solely as something related to religion but as a source of meaning and purpose in life. Religious/spiritual beliefs can play a critical role in how individuals experience and cope with depressive symptoms (52). Religious involvement is especially relevant in stress-related or situational depression, as it may influence how individuals interpret and respond to adverse events. Additionally, evidence from randomized clinical trials suggests that religious participation and spiritually integrated interventions can contribute to reductions in depressive symptoms and promote psychological resilience (52).

Our findings showed a surprising pattern of similar positive and negative religious coping changes following the intervention. Traditionally, positive religious coping has been associated with a secure relationship with God, spiritual growth, and psychological resilience, while negative religious coping has been linked to greater emotional distress and poorer wellbeing outcomes (53). One possible explanation for our findings is that negative religious coping may reflect a spiritual struggle that, although distressing, can also act as a transformative agent for psychological growth and meaning-making (54). Spiritual struggle may (at least for some people) represent a stage in the broader spiritual and psychological development process. This interpretation may be particularly relevant given that religious coping styles may not always fit neatly into positive versus negative dichotomies (55). Although much work addresses the role of spirituality in shaping psychological processes (56, 57), an open question for future research is whether a new understanding of spirituality necessarily signals an improvement in participants’ psychological functioning.

Although it can be challenging to pinpoint the exact mechanisms underlying a multidimensional intervention and further work is needed to explore different possibilities, we theorize that the mechanisms driving change in the Flourishing Intervention are multifactorial. One possible explanation for the pattern of findings observed in this study is that positive activities increase positive emotions, thoughts, behaviors, and satisfaction of needs. Engaging in positive activities helps individuals interpret events more optimistically, promoting a positive feedback loop, which can increase satisfaction over time (58). This idea aligns with the broaden-and-build theory of positive emotions (59, 60). In particular, Fredrickson (60) proposed an upward spiral theory of lifestyle change as a framework for understanding the mechanisms by which positive emotions might alter health behaviors. As positive affect is experienced during a new health behavior, the upward spiral theory proposes that it creates unconscious motives for that activity, which become stronger over time as advantageous resources, both biological and psychological, increasingly support them. Over time, these changes support the development of personal resources – psychological, cognitive, social, and physical – that can contribute to ongoing wellbeing (59). The Flourishing Intervention may have a similar upward spiral effect throughout the intervention, which might explain why post-intervention improvements were observed across a range of whole person functioning outcomes.

### 4.1 Practical and clinical implications

Our study has potential implications for the promotion of human flourishing. We found that the Flourishing Intervention was feasible and highly accepted, suggesting that it may be incorporated in primary care settings (61). However, it can also be used as a complementary strategy to conventional mental health treatment provided by community mental health centers. Because many individuals with depression do not pursue treatment, there is a need for innovative interventions targeting individuals who are unlikely to seek formal treatment due to stigma, lack of resources, or accessibility challenges. These interventions can be structured to reach those who may require support but would not typically seek help in a formal healthcare setting (61). The Flourishing Intervention proved feasible for use with individuals experiencing depressive symptoms in Brazil. Given the similarities between Brazil and other developing countries, especially those with pervasive social-structural vulnerabilities (e.g., South Africa), the intervention may have broader applicability in global healthcare contexts.

Due to its broader scope and its principal focus on promoting a person’s wellbeing, the Flourishing Intervention may be less stigmatizing for people who may be concerned about conventional treatment options and diagnostic labels (62). It may also be appealing to those who are struggling with depressive symptoms that are secondary to other concerns that are potentially influencing their treatment decisions. For example, we found that many participants were motivated to participate in the Flourishing Intervention because of the physical health problems they were experiencing, even though they endorsed moderate to moderately severe depressive symptoms. If some people are motivated to participate in this kind of intervention based on other primary concerns (e.g., religious/spiritual struggles) that have known linkages to depression (63), the Flourishing Intervention could be beneficial to a range of individuals who may not necessarily be contemplating or actively seeking treatment because of their depressive symptoms.

Another noteworthy consideration is that any healthcare professional can deliver the Flourishing Intervention. This flexibility increases feasibility and broadens the possible avenues for delivering the intervention. For example, professionals from various healthcare professions could facilitate the Flourishing Intervention, provided they undergo appropriate training. The online format of the Flourishing Intervention may also promote greater accessibility for individuals who find it difficult to travel to a particular location due to health or financial reasons.

### 4.2 Limitations

Our study has limitations that should be considered. A key limitation of this study is the absence of a control group, which restricts our ability to make definitive causal inferences. As no control group was used, our results relied on pre-post comparisons for the treatment group. Several factors, such as social interactions during group sessions, the therapeutic alliance with the intervention providers, other services or support (e.g., individual psychotherapy) that participants may have received, or even the placebo effect, could have acted as confounding variables. Our findings may also be biased by non-adherence to the intervention and the differences observed in age between those who completed and dropped out of the intervention. However, the convergence of findings across quantitative and qualitative data provides preliminary evidence supporting the feasibility and acceptability of the Flourishing Intervention. Randomized controlled trials are needed to establish whether the Flourishing Intervention is causally related to the outcomes examined.

Our recruitment was primarily carried out through advertisements on the social media platforms of university hospitals. This recruitment approach may have attracted individuals with higher levels of education, higher incomes, and internet access. Outcomes were assessed using self-report measures, so it is possible the results may have been affected by self-report biases (e.g., socially desirable responding).

While the Flourishing Intervention is multidimensional by design, one drawback of this approach is that it can be challenging to pinpoint specific mechanisms responsible for the observed effects. Although the 48.5% completion rate observed in this study may limit the generalizability of the quantitative findings, it is consistent with attrition rates reported in online interventions targeting individuals with depressive symptoms. Previous research has shown that self-guided web-based interventions can experience attrition rates as high as 45% (64), underscoring the challenges of maintaining engagement in remote intervention studies. Our intervention was delivered in a group-based online format with active facilitation by trained health professionals, which may have played a role in retaining participants who completed the intervention. We used strategies to try to minimize attrition (e.g., using text messages or phone calls to maintain contact with participants and follow-up in cases of missed sessions or delayed responses). However, other strategies could be explored to improve participant retention in future research involving the Flourishing Intervention.

### 4.3 Conclusion

The Flourishing Intervention shows promise as a practical approach for improving wellbeing. Our findings support the idea that a multidimensional intervention focused on promoting a whole-person functioning can alleviate depressive symptoms among adults. Lessons learned from this study can help refine intervention procedures, shape future research examining the effects of the Flourishing Intervention using a controlled design and contribute more broadly to promoting an expanded view of flourishing.

## Data Availability

The raw data supporting the conclusions of this article will be made available by the authors, without undue reservation.
