# Supplementary material for: Promoting the wellbeing of the whole person: a within-subjects mixed-methods study exploring the effects of the flourishing intervention among individuals with depressive symptoms
Source: Front Psychiatry. 2025 Jun 19;16:1532843. doi: 10.3389/fpsyt.2025.1532843 (PMC12223424; doi:10.3389/fpsyt.2025.1532843)
Supplement: Supplementary file 2 [file Table2.docx]

**Supplementary Materials**

**Focus group guiding questions for the qualitative interviews**

| ***Overview***  I am interested in knowing why you agreed to participate in the intervention?  What did you initially expect from the intervention?  At the end of the intervention, were your expectations met?  ***Process of Change***  What changes in yourself did you feel during the study (and at the end of the intervention)?  Do you perceive benefits in participating in the intervention?  ***Satisfaction and Engagement***  Which elements of the intervention did you like the most?  What did you think of the online format of the intervention?  What suggestions would you provide to improve the intervention?  What is your opinion about the scheduled time and duration of the intervention?  After how many sessions did you feel more comfortable sharing your opinions?  What did you think of the approach taken by the providers? |
| --- |
